# Molecular study on *Pasteurella multocida* and *Mannheimia granulomatis* from Kenyan Camels (*Camelus dromedarius*)

**DOI:** 10.1186/s12917-017-1189-y

**Published:** 2017-08-22

**Authors:** Ilona V. Gluecks, Astrid Bethe, Mario Younan, Christa Ewers

**Affiliations:** 1 Vétérinaires sans Frontières Suisse, Nairobi, Kenya; 2 Institute of Microbiology and Epizootics, Centre for Infection Medicine, Free University Berlin, Berlin, Germany; 3 Vétérinaires sans Frontières Germany, Nairobi, Kenya; 4 Institute of Hygiene and Infectious Diseases of Animals, Justus-Liebig University Giessen, Giessen, Germany

**Keywords:** Pasteurellaceae, *Pasteurella multocida*, *Mannheimia*, Camels, *Camelus dromedarius*, Haemorrhagic Septicaemia

## Abstract

**Background:**

Outbreaks of a Haemorrhagic Septicaemia (HS) like disease causing large mortalities in camels (*Camelus dromedarius*) in Asia and in Africa have been reported since 1890. Yet the aetiology of this condition remains elusive. This study is the first to apply state of the art molecular methods to shed light on the nasopharyngeal carrier state of Pasteurellaceae in camels. The study focused on HS causing *Pasteurella multocida* capsular types B and E. Other Pasteurellaceae, implicated in common respiratory infections of animals, were also investigated.

**Methods:**

In 2007 and 2008, 388 nasopharyngeal swabs were collected at 12 locations in North Kenya from 246 clinically healthy camels in 81 herds that had been affected by HS-like disease. Swabs were used to cultivate bacteria on blood agar and to extract DNA for subsequent PCR analysis targeting *P. multocida* and *Mannheimia*-specific gene sequences.

**Results:**

Forty-five samples were positive for *P. multocida* genes *kmt* and *psl* and for the *P. multocida* Haemorrhagic Septicaemia (HS) specific sequences KTSP61/KTT72 but lacked HS-associated capsular type B and E genes *capB* and *capE*. This indicates circulation of HS strains in camels that lack established capsular types. Sequence analysis of the partial 16S rRNA gene identified 17 nasal swab isolates as 99% identical with *Mannheimia granulomatis*, demonstrating a hitherto unrecognised active carrier state for *M. granulomatis* or a closely related *Mannheimia* sp. in camels.

**Conclusions:**

The findings of this study provide evidence for the presence of acapsular *P. multocida* or of hitherto unknown capsular types of *P. multocida* in camels, closely related to *P. multocida* strains causing HS in bovines. Further isolations and molecular studies of camelid *P. multocida* from healthy carriers and from HS-like disease in camels are necessary to provide conclusive answers. This paper is the first report on the isolation of *M. granulomatis* or a closely related new *Mannheimia* species from camelids.

## Background

*Pasteurella multocida* capsular types B and E are the specific cause of seasonal outbreaks of Haemorrhagic Septicaemia (HS) in tropical cattle and buffaloes [1–3]. The veterinary literature provides a long record of an HS-like disease causing significant mortality in camels [4–8]. Yet the aetiology of HS in camels remains elusive [1, 3, 9]. Previous reports on HS-like disease in camels often failed to isolate the pathogen or provided only limited information on phenotypical characteristics of the isolates [9]. Attempts to infect camels with highly virulent *Pasteurella multocida* capsular type B strains produced only very mild clinical symptoms that resolved completely within 3 days [9].

A respiratory disease in Ethiopian camels caused by *Pasteurella (Mannheimia) haemolytica* has been described by Bekele [10]. This pilot study is the first of its’ kind to use state of the art molecular methods for investigating Pasteurellaceae in camels.

## Methods

In 2007 and 2008, 388 nasopharyngeal swabs were collected at 12 locations in North Kenya from 246 clinically healthy camels in 81 herds that had reportedly been affected by outbreaks of HS-like disease. Flocculated swabs (FLOQSwabs®) were used for DNA extraction while swabs in Amies transport medium (Sterilin®) were used for standard bacteriological investigation. Bacteriological examination and extraction of DNA was carried out at a laboratory in Nairobi (Analabs Ltd.). DNA eluates from 341 swabs and 19 isolated *Pasteurella*-like cultures were transferred to the Institute for Microbiology and Epizootics at Freie Universität Berlin (IMT/FUB) in Germany. At the IMT/FUB, DNA was extracted from 19 culture isolates, two of which did not belong to the Pasteurellaceae based on PCR results. Seventeen isolates were subsequently selected for biochemical testing to differentiate *M. granulomatis* from other *Mannheimia* spp. according to Ewers et al. (2004) [11]. Three hundred five samples with positive reaction in the 16S rRNA gene PCR, as well as, heat denatured DNA from bacterial cultures were investigated for the presence of *P. multocida*- and *M. haemolytica*- specific DNA sequences. For *P. multocida* the specific sequences tested were *kmt, psl,* and KTSP61/KTT72. Samples that proofed positive in one of these PCRs were further tested for capsular genes *capA*, *capB*, *capD*, *capE*, and *capF*, and for virulence-associated genes *toxA*, *ptfA*, *pfhA oma87*, *ompH*, *hgbA*, *hgbB*, *exbB*/*tonB*, *tbpA*, *nanB*, and *nanH*. To detect pathogenic *Mannheimia* spp., we screened for the leukotoxin (*lktA*) and the outer membrane protein (*pomA*) genes. All PCRs, except for the *P. multocida* capsular gene PCR, which was performed as a multiplex PCR, were conducted as single PCRs according to previously published protocols [12–18].

## Results

The species *P. multocida* could not be isolated from 312 nasal swabs cultured on Blood Agar. Of the 305 DNA eluates containing sufficient quantities of DNA to undergo molecular characterization, 60 were positive for at least one of the *P. multocida* species-specific sequences tested by PCR. Forty-five samples gave a positive result for the HS- associated sequence KTSP61/KTT72, known to be present in *P. multocida* capsular type B strains. Neither *capB* nor *capE* gene, encoding HS-associated capsular types B and E, were detected in the sample material. Blast search of 18 KTSP61/KTT72 sequences [19] matched with high scores (98.8–99%) to nucleotide sequences submitted under GenBank accession numbers AF016260 (*P. multocida* unknown protein 1 gene, partial cds and unknown protein 2 gene, complete cds), AY948545.1 (*P. multocida* HS-B specific genomic sequence) and AJ421513.1 (*P. multocida* DNA fragment specific for HS). *CapA* and *capD* genes encoding for *P. multocida* capsular types A and D were identified in five and in one sample, respectively. Screening for adhesion-related genes *ptfA* and *pfhA* was positive in 15 and 2 samples, respectively. Outer membrane protein gene *ompH* was detected in 10, *oma87* in 14 samples. While *toxA*, encoding the dermonecrotoxin, was not found in any sample, iron acquisition-related genes were present as follows: *hgbA* (*n* = 12), *hgbB* (*n* = 11), *exbB*/*tonB* (*n* = 1). Neuraminidase encoding genes *nanB* and *nanH* were present in 9 and 11 samples, respectively.

The biochemical profiles of the 17 *Mannheimia* spp. isolates characterised phenotypically were inconsistent and differed from the biochemical profile described for *M. granulomatis* [20], namely Sorbitol: pos.; α-Fucosidase: neg.; β-Galactosidase: variable. Of the 305 eluates tested, none was positive for *Mannheimia* spp. associated sequences *lktA* or *pomA*. According to the 16S rRNA sequence analysis performed on 19 cultures from the nasopharynx of healthy camels, 17 sequences showed closest relatedness (98–99%) to sequences from *M. granulomatis*, previously classified as *P. granulomatis*, Bisgaard taxon 20 and *P*. *haemolytica* biogroup 3 J [21, 22].

## Discussion

This study investigated the active (nasopharyngeal) carrier state for Pasteurellaceae in Kenyan camels. Importance of the latent carrier state in the epidemiology of Pasteurellosis was reiterated by Dziva et al. (2008) [23].

The fact that no *P. multocida* capsular type B or E specific DNA sequences were identified in this study may indicate the presence of non-capsulated strains or the emergence of a hitherto unknown capsular type of *P. multocida* in camels. According to the OIE Terrestrial Manual [24] vaccines against Haemorrhagic Septicaemia in cattle and buffaloes must be based on local isolates that represent the prevalent serotype; seed cultures for the production of HS vaccines should contain capsulated organisms. Based on the results of this study the indiscriminate use of vaccines based on HS causing *P. multocida* isolates from cattle and buffaloes cannot be recommended for the prevention of HS-like disease in camels. - This is the first molecular study to confirm the presence of *P. multocida* capsular types A and D specific DNA sequences in the nasopharynx of healthy Kenyan carrier camels, albeit at low frequency. Both capsular types have been reported in camels previously [25] based on phenotypic characterisation. Failure to culture *P. multocida* in this study is possibly related to the fastidiousness of the species which does not withstand cold chain transport of several days [26].

The *lktA* or *pomA* sequences do not occur regularly in all *Mannheimia* species or strains [27, 28], hence negative findings do not rule out presence of *Mannheimia* spp., but *lktA*-negative *M. haemolytica* strains are reported to be less virulent. In this study the most common Pasteurellaceae species cultured from the nasopharynx of healthy carrier camels in North Kenya and identified by 16S rRNA sequence analysis was a *Mannheimia* sp. with 98% to 99% sequence identity to *M. granulomatis*. Only more recently has *M. granulomatis* been recognised as a significant pathogen in domestic and wild ruminants [29–31]. Phenotypic characterization of Pasteurellaceae species is of limited value [32] and published phenotypic characteristics for *M. granulomatis* are based on a limited number of bovine, leprine and deer strains [2, 21, 22, 30]. Hence it is to be expected that biochemical reactions of Kenyan camelid *M. granulomatis* strains differ from those described for *M. granulomatis* under http://www.bacterio.net/mannheimia.html/. Comparison of our results with a previous communication on involvement of *P.* (*M.*) *haemolytica* in respiratory disease in Ethiopian camels [10] is also limited, because the methodology used there would not have permitted a differentiation between *M. granulomatis, M.* (*Pasteurella*) *haemolytica* and other *Mannheima* spp. The possibility that this respiratory pathogen isolated from Ethiopian camels [10] may in fact have been *M. granulomatis* or a new *Mannheimia* species very closely related to *M. granulomatis* cannot entirely be ruled out*.*

## Conclusions

This study has documented the carrier state for acapsular *P. multocida* or unknown capsular types of *P. multocida*, closely related to the *P. multocida* strains causing HS in cattle and buffaloes, in healthy camels. At the same time the study found no evidence for the presence of *P. multocida* capsular types B and E or their specific DNA sequences in healthy camels in North Kenya. Further isolations and molecular studies of camelid *P. multocida* from healthy carriers and from HS-like disease in camels are necessary to provide conclusive answers. To our best knowledge this is the first report on the isolation of *M. granulomatis* or a closely related new *Mannheimia* species from camelids.

## Abbreviations

DNA: Deoxyribonucleic acid
HS: Haemorrhagic Septicaemia
IMT/FUB: Institute for Microbiology and Epizootics at Freie Universität Berlin
PCR: Polymerase Chain Reaction
RNA: Ribonucleic acid
rRNA: Ribosomal ribonucleic acid
